# Thigh muscle metabolic response is linked to feed efficiency and meat characteristics in slow-growing chicken

**DOI:** 10.1016/j.psj.2023.102741

**Published:** 2023-04-23

**Authors:** Pramin Kaewsatuan, Chotima Poompramun, Satoshi Kubota, Jirawat Yongsawatdigul, Wittawat Molee, Pekka Uimari, Amonrat Molee

**Affiliations:** ⁎ School of Animal Technology and Innovation, Institute of Agricultural Technology, Suranaree University of Technology, Nakhon Ratchasima 30000, Thailand; † School of Food Technology, Institute of Agricultural Technology, Suranaree University of Technology, Nakhon Ratchasima 30000, Thailand; ‡ Department of Agricultural Sciences, Faculty of Agriculture and Forestry, University of Helsinki, Helsinki 00790, Finland

**Keywords:** feed efficiency, meat characteristics, hub proteins, WGCNA, slow-growing chicken

## Abstract

The Korat chicken (**KR**) is a slow-growing Thai chicken breed with relatively poor feed efficiency (**FE**) but very tasty meat with high protein and low fat contents, and a unique texture. To enhance the competitiveness of KR, its FE should be improved. However, selecting for FE has an unknown effect on meat characteristics. Thus, understanding the genetic basis underlying FE traits and meat characteristics is needed. In this study, 75 male KR birds were raised up to 10 wk of age. For each bird, the feed conversion ratio (**FCR**), residual feed intake (**RFI**), and physicochemical properties, flavor precursors, and biological compounds in the thigh meat were evaluated. At 10 wk of age, thigh muscle samples from 6 birds (3 with high FCR and 3 with low FCR values) were selected, and their proteomes were investigated using a label-free proteomic method. Weighted gene coexpression network analysis (**WGCNA**) was used to screen the key protein modules and pathways. The WGCNA results revealed that FE and meat characteristics significantly correlated with the same protein module. However, the correlation was unfavorable; improving FE may result in a decrease in meat quality through the alteration in biological processes including glycolysis/gluconeogenesis, metabolic pathway, carbon metabolism, biosynthesis of amino acids, pyruvate metabolism, and protein processing in the endoplasmic reticulum. The hub proteins of the significant module (TNNT1, TNNT3, TNNI2, TNNC2, MYLPF, MYH10, GADPH, PGK1, LDHA, and GPI) were also identified to be associated with energy metabolism, and muscle growth and development. Given that the same proteins and pathways are present in FE and meat characteristics but in opposite directions, selection practices for KR should simultaneously consider both trait groups to maintain the high meat quality of KR while improving FE.

## INTRODUCTION

The Korat chicken (**KR**) is a crossbreed between Thai indigenous line males (Leung Hang Khao: **LHK**) and synthetic Suranaree University of Technology (**SUT**) line females (Poompramun et al., 2021b). KR is known for its healthy meat, with rich flavor and unique texture that are valued by consumers (Katemala et al., 2021). However, poor feed efficiency (**FE**) of KR increases production costs and reduces the competitiveness of KR against other commercial chicken commonly used in Thailand. To improve profitability and competitiveness, a balance between FE and meat characteristics is an important breeding goal for KR.

Traditionally, FE is measured as a feed conversion ratio (**FCR**); a ratio of feed intake (**FI**) to body weight gain (**BWG**). Selecting for FCR more efficiently improves the numerator trait (FI) compared with the denominator trait (BWG), a well-known limitation of FCR that causes nonlinear selection pressure (Gunsett, 1984). Residual feed intake (**RFI**) is another commonly used FE measurement; it is the difference between observed and expected feed intake based on requirements for maintenance and growth (Koch et al., 1963). Due to its low genetic correlation with production traits and a moderate correlation with FCR and FI, RFI is considered the most appropriate trait for the genetic improvement of energy efficiency in poultry (Willems et al., 2013; Xu et al., 2016). However, improving FE potentially causes a decrease in meat quality and reduces consumer acceptance of meat (Zhou et al., 2015). In addition, FE is age dependent in chicken (Arthur et al., 2001; Berry and Pryce, 2014) due to developmental processes and maturity of the physiological functions of the tissues (Aggrey et al., 2010; Ravindran and Abdollahi, 2021).

Previous skeletal muscle transcriptomic analysis in chicken has indicated that FE may be related to nucleotide sugar biosynthesis, glycogen metabolism, and lipid uptake and transport (Abasht et al., 2019). Our previous transcriptomic analysis with KR chicken indicated that nucleotide metabolism, fatty acid metabolic process, and oxidative stress play key roles in regulating both FE and the quality of thigh meat (Poompramun et al., 2021a). Thus, improving FE can have a negative impact on meat texture and the nutritional value of meat via activating the accumulation of biochemical compounds and flavor precursors. Although the molecular mechanisms have been extensively investigated at the transcriptome level (Poompramun et al., 2021a), understanding is lacking on how the FE and thigh muscle meat quality of this slow-growing chicken breed are linked at the proteomics level. Overall, a better understanding of how FE affects meat characteristics is crucial to avoiding any unfavorable effects of improving FE on the quality and characteristics of KR thigh meat.

In this study, we use label-free proteomics to profile the thigh muscle proteome of KR with either high or low FE and to identify key proteins and molecular pathways related to both FE and meat characteristics. Our main hypotheses are: 1) the regulation of FCR and RFI depend on several molecular and physiological mechanisms, 2) a negative relationship exists between FE and meat characteristics, and 3) the molecular and physiological determinants of FCR and RFI are age dependent. New information gained can be used in selection strategies to improve FE while retaining the excellent meat characteristics of KR.

## MATERIALS AND METHODS

### Ethics Statement

The experiment was carried out at Suranaree University of Technology's experimental farm. All experimental procedures were approved by the Ethics Committee on Animal Use constituted by the Suranaree University of Technology in Nakhon Ratchasima, Thailand (U1-02631-2559).

### Experimental Animals and Tissue Collection

Seventy-five male Korat chicken were used in this experiment. The birds were produced by mating individuals with the highest body weights together and by mating individuals with the lowest body weights together. At hatching, the birds were sexed using the vent sexing method, individually weighted, wing banded, and vaccinated against Marek's disease. Subsequently, they were vaccinated following the recommendation of the Department of Livestock Development, Thailand. All birds were individually raised in the same conditions in 63 × 125 × 63 cm cages covered with rice hulls. The diet of birds 0 to 3 wk of age included 21% protein (a starter diet), 19% protein for birds 4 to 6 wk of age (a grower diet), and 17%% protein for birds 7 to 10 wk of age (a finisher diet). Birds were fed ad libitum and water was freely available in each cage through nipple drinkers.

At 10 wk of age, all birds were transported to the slaughterhouse, rested for 30 min, and electrocuted before having their necks cut and before being bled and plucked. The carcasses were manually eviscerated and washed before being stored in a cold room (4°C). A piece of thigh muscle was collected from each bird, snap frozen in liquid nitrogen, and stored at −80°C until further proteome analyses.

### Feed Efficiency

Feed efficiency was determined using 2 measurements: FCR and RFI. The FCR was calculated for each bird i and week k as FCR_ik_ = FI_ik_ /BWG_ik_, where FI_ik_ is the total feed intake of bird i from hatch to week k and BWG_ik_ is the BWG of the same bird for the same period. Only FCRs for wk 2, 4, 6, 8, and 10 were used in the statistical analysis.

The RFIs were first calculated for different weeks using the following formula:

$$\text{RE}S_{\text{ij}}=FI_{\text{ij}}-\left(b_{0}+b_{1}\text{MM}E_{\text{ij}}+b_{2}\text{BW}G_{\text{ij}}\right)$$

where FI_ij_ is the total feed intake of bird i during week j, MMW_ij_ is the metabolic weight of bird i estimated from mean body weights at weeks j and j − 1, ${\left(\frac{BW_{\text{ij}}+BW_{\text{ij}-1}}{2}\right)}^{0.75}$, BWG_ij_ is the body weight gain between weeks j and j − 1, b_0_ is the intercept, and b_1_ and b_2_ are partial regression coefficients. The weekly RFIs were combined for statistical analyses, that is, for bird i, the RFI for week k is $\text{RF}I_{\text{ik}}=\sum_{j=1}^{k}\text{RE}S_{\text{ij}}$. Again, in the statistical analyses, only RFIs for wk 2, 4, 6, 8, and 10 were used.

In the present study, 6 male KR exhibiting the most extreme FCRs at 10 wk of age (FCR_10_) were chosen for further proteomic analyses: 3 with the highest FCRs (FCR_10_ = 3.33, 3.34, and 3.36) and 3 with the lowest FCRs (FCR_10_ = 1.83, 1.98, and 1.99). These groups are later referred to as the low-FE and high-FE groups, respectively. The studied birds were the same as in our previous gene expression study of the KR thigh muscle (Poompramun et al., 2021a).

### Meat Characteristic Measurements and Chemical Analyses

For meat quality measurement, thigh meat samples were collected 24 h after chilling. We consider only those meat characteristics that relate to thigh muscle texture and flavor including physicochemical properties, nucleotide contents, and biomolecules. For more details concerning sample collection, preparation, and measurements, see the previous study by Poompramun et al. (2021a).

#### Physicochemical Properties

The thigh meat's ultimate pH (**pHu**) was evaluated in triplicate with a portable pH meter (pHCore-kit, Satorius, Goettingen, Germany). Prior to conducting measurements, the pH meter was calibrated using standardized buffers (pH 4.01 and 7.00) at room temperature. Water-holding capacity (**WHC**) was determined with a 5-g sample of the thigh muscle. The samples were weighted, wrapped between 3 pieces of filter paper (Whatman No. 4; Whatman Inc., Clifton, NJ), and centrifuged at 3,000 × *g* for 20 min. To evaluate drip loss (**DL**), the individual thigh samples were cut into 1.0 × 2.0 × 0.5 cm pieces with an approximate weight of 4 to 5 g per piece. These were wrapped in absorbent pads and placed in polyethylene bags before being stored at 4°C for 24 h, then reweighed to calculate the percentage of drip loss.

#### Nucleotide Content Analysis

From each thigh sample, a 5-g sample was mixed with 30 mL of 0.75 M perchloric acid, homogenized for 30 s, and centrifuged at 2,000 × *g* (Thermo Fisher Scientific, Waltham, MA). The extracted nucleotides were analyzed using HPLC (HP 1260; Agilent Technologies, Santa Clara, CA). The concentrations of guanosine monophosphate (**GMP**), inosine monophosphate (**IMP**), adenosine monophosphate (**AMP**), and inosine (**Ino**) were quantified by comparing the area under the peak with the external standards (Sigma-Aldrich Co., St. Louis, MO).

#### Biomolecular Changes

Fourier transform infrared (**FTIR**) microspectroscopy was used to reveal any changes in biomolecule profiles related to FE. The individual thigh samples were chopped into pieces and placed in aluminum foil boxes. All samples were snap-frozen at −80°C for 24 h and dehydrated for 24 h in a laboratory freeze-dryer, after which the freeze-dried samples were pulverized into powder. All FTIR spectra were acquired in attenuated total reflectance (**ATR**) mode with a single reflection ATR sampling module coupled with OPUS7.2 software (Bruker Optics Ltd., Ettlingen, Germany). The spectra were recorded in the mid-infrared range of 4,000 to 600 cm^−1^ with a resolution of 4 cm^−1^ and averaging 64 coadded scans (Bruker Optics Ltd., Ettlingen, Germany). The peak areas of integration of each biomolecule were derived using second-derivative processing at the spectral regions from 3,000 to 900 cm^−1^, which were assigned to C–H stretching (lipid, 3,000−2,800 cm^−1^), >C=O stretching (ester carbonyl of phospholipids, 1,743 cm^−1^), C=O stretching (amide I, 1,700−1,600 cm^−1^), C–N stretching + N–H bending coupled out of face (amide II, 1,600−1,500 cm^−1^), C–N stretching + N–H bending coupled in of face (amide III, 1,338 cm^−1^), C–H bending (1,450 and 1,380 cm^−1^), and C–O–C, C–O dominated by ring vibrations of carbohydrates C–O–P, P–O–P (carbohydrate and glycogen; 1,250−900 cm^−1^).

### Statistical Analysis

The Student *t* test was used to compare the means of FE, physicochemical properties, nucleotide contents, and biomolecules of the thigh meat between the low-FE and high-FE groups (SPSS 20.0 statistical software; SPSS Inc., Chicago, IL).

### Protein Analysis

Thigh muscle tissue from 6 samples were freeze-dried and ground into a fine powder. Samples were lysed in denaturing buffer (50 mM ammonium bicarbonate containing 8 M urea: **AMBIC**, Sigma-Aldrich, St. Louis, MO) using the sonication method. The lysates were clarified by centrifugation at 20,000 × *g* for 10 min at 4°C. The collected proteins were diluted with 50 mM AMBIC (final concentration of 1.5 M urea). Protein quantifications were then determined with the Pierce BCA Protein Assay kit (Thermo Fisher Scientific, Waltham, MA). Proteins were reduced with dithiothreitol (a final concentration of 5 mM) for 20 min at 50 to 60°C. Then, iodoacetamide (final concentration of 15 mM) was added to the protein samples and incubated for 20 min at room temperature in a dark environment, followed by protein digestion with 2 µg of trypsin at 37°C for 24 h.

Peptide mixtures were analyzed using a Q Exactive Hybrid Quadrupole-Orbitrap Mass Spectrometer (Thermo Fisher Scientific, Waltham, MA) at the Proteomics Unit core facility, University of Helsinki, Finland. The peptides were loaded onto a C18 reverse-phase column on an 80-min gradient. MS data were operated using a data-dependent acquisition (**DDA**) mode and higher-energy collisional dissociation (**HCD**) for mass fragmentation. One technical replicate from each 6 samples was combined and analyzed for the database search.

Raw MS files from Orbitrap mass spectrometry were processed using version 1.6.5.0 of MaxQuant (Cox and Mann, 2008). The mass spectra were annotated against the Uniprot *Gallus gallus* database (34,925 total entries, downloaded from https://www.uniprot.org, January 2019 version). Two missed cleavages were allowed for trypsin specificity. Data searches were conducted with variable modifications of oxidation (M) and acetyl (protein N-term), and carbamidomethylation was set as a fixed modification. The initial mass precursor tolerance was set to 20 ppm and 6 ppm in the first search and main search, respectively. Furthermore, fragment (MS/MS) mass deviation was set to 20 ppm. The false discovery rates (**FDR**) for peptide and protein identification were set to 0.01. Protein abundance was defined by the normalized spectral protein intensity (LFQ intensity). Prior to bioinformatics analysis, the data were normalized, and missing values were imputed. The experimental proteomic data sets in the current study were deposited in ProteomeXchange Consortium via the PRIDE (https://www.ebi.ac.uk/pride/) partner repository, project accession: PXD039718 (Reviewer account details: Username: reviewer_pxd039718@ebi.ac.uk; Password: YJhXK7Yn).

### Protein Network Construction and Module Identification

Bioinformatic analyses, that is, protein networks construction, highly correlated proteins cluster (module) identification, and their further analysis were based on Wight Gene Co-expression Network Analysis (**WGCNA**), available in the R package (Langfelder and Horvath, 2008).

To specify an unsigned network, a weighted adjacency matrix was formed based on absolute values of the pairwise Pearson's correlation coefficients among all proteins. The soft threshold power (β) was set to 9 to reach the scale-free topology criterion (*R*^2^ = 0.80). The topological overlap measure (**TOM**) and the corresponding dissimilarity (1 − TOM) were calculated using the adjacency matrix. The modules of coexpressed proteins were identified by the Dynamic Tree Cut algorithm. The minimum size of the module was set to 30 proteins. Modules that were very similar were merged (height cut-off of 0.15 in the dendrogram). Finally, each module was labeled with different colors, and proteins that did not belong to any of the modules were grouped together to form their own module (gray).

### Module–Trait Relationship

The module eigenproteins (**ME**), defined as the first principal components of the modules (Langfelder and Horvath, 2008), were used to study the relationships between the modules and FE and meat characteristics including chemical compounds. The relationships were based on Pearson's correlations and only modules that were significantly (*P* < 0.05) correlated with FE and meat characteristics were used in the functional enrichment analysis.

### Functional Enrichment Analysis of Proteins

To gain insight into the potential biological function of the identified proteins, all proteins from the selected module were subject to the gene ontology (**GO**) function and Kyoto Encyclopedia of Genes and Genomes (**KEGG**) pathway enrichment analysis using STRING (version 11.5, http://string-db.org, Szklarczyk et al., 2015). Functional enrichment was carried out in 3 GO categories: biological process (**BP**), molecular function (**MF**), and cellular component (**CC**). The GO results were visualized using the R package “ggplot2” (version 3.3.5, https://ggplot2.tidyverse.org/). Enriched GO proteins and KEGG pathways with FDR-adjusted (Benjamini-Hochberg method) *P* values <0.05 were considered statistically significant.

### Hub Protein Identification

Hub proteins were characterized as proteins with the highest connectivity. Hub proteins were identified based on their protein significance (|**PS**| ≥ 0.6), corresponding to the absolute value of the correlation between the protein expression profile and the traits of interest, and module membership (|**MM**| ≥ 0.7), defined as the correlation between the MEs and its protein abundance profile. Subsequently, the hub proteins were provided as input into Cytohubba, a Cytoscape plugin (version 3.6.1, https://cytoscape.org/), to identify the highest linkage hub proteins in the network based on the Maximal Clique Centrality (**MCC**) algorithm (Chen et al., 2009).

## RESULTS

### Phenotypic Data of Korat Chickens

The means and standard errors of FE, flavor precursors (nucleotides) and physiochemical properties for the low-FE and high-FE groups are presented in Table 1. As expected, a significant difference in FCR or RFI was observed between the groups. Additionally, the differences in WHC and nucleotides were significant. Also, the integral area of amide I and C–H bending was higher in the high-FE group compared with the low-FE group (Table 2).

**Table 1:** Means (±SE) of feed efficiency (FE), physiochemical properties, and nucleotides of thigh meat in the high-FE and low-FE chicken groups.

Parameters	Low-FE	High-FE	*P* value
Feed conversion ratio at 10 wk, FCR_10_	3.34 ± 0.01	1.93 ± 0.05	<0.01⁎⁎
Residual feed intake at 10 wk, RFI_10_	158.13 ± 162.52	−724.02 ± 78.98	<0.01⁎⁎
Bodyweight gain, BWG (g)	1138.93 ± 49.30	1638.08 ± 84.60	<0.01⁎⁎
Total feed intake from 1 wk. to 10 wk, FI (g)	3807.38 ± 168.30	3173.07 ± 209.25	0.08
Ultimate pH, pH24	6.15 ± 0.26	6.29 ± 0.10	0.65
Water-holding capacity, WHC (%)	89.65 ± 1.60	78.68 ± 0.90	<0.01⁎⁎
Drip loss, DL (%)	6.65 ± 1.33	11.51 ± 1.57	0.08
Guanosine monophosphate, GMP (mg/g)	0.12 ± 0.02	0.12 ± 0.03	0.92
Inosine monophosphate, IMP (mg/g)	4.89 ± 0.21	3.93 ± 0.61	0.21
Adenosine monophosphate, AMP (mg/g)	0.08 ± 0.01	0.12 ± 0.01	0.03⁎
Inosine (mg/g)	0.31 ± 0.02	0.66 ± 0.06	<0.01⁎⁎

⁎ *P* < 0.05

⁎⁎ *P* < 0.01

**Table 2:** Means (±SE) of the integrated area of biomolecules based on synchrotron radiation-Fourier transform infrared (SR-FTIR) microspectroscopy in the high-FE and low-FE chicken groups.

Biomolecules (wavenumbers)	Low-FE	High-FE	*P* value
Lipids (3,000−2,800 cm^−1^)	0.041 ± 0.004	0.053 ± 0.008	0.30
Ester carbonyl of phospholipids (1,743 cm^−1^)	0.014 ± 0.003	0.014 ± 0.004	0.54
Amide I (1,700−1,600 cm^−1^)	0.050 ± 0.001	0.062 ± 0.003	<0.01⁎⁎
Amide II (1,600−1,500 cm^−1^)	0.040 ± 0.001	0.040 ± 0.002	0.96
Amide III (1,338 cm^−1^)	0.0003 ± 0.000	0.0005 ± 0.000	0.43
C–H bending (1,450, 1,380 cm^−1^)	0.031 ± 0.002	0.047 ± 0.001	<0.01⁎⁎
Carbohydrate and glycogen (1,250−900 cm^−1^)	0.025 ± 0.002	0.036 ± 0.005	0.15

⁎ *P* < 0.05

⁎⁎ *P* < 0.01

### Coexpressed Protein Network Construction and Key Module Identification

A total of 904 proteins were detected from 6 thigh muscle tissue samples of KR. Their abundance profiles are presented in the Supplementary Table 1. After filtering, 313 proteins remained and were used to explore the association between the proteomic profiles and the FE and meat characteristic traits using WGCNA. Four modules were identified (blue, brown, turquoise, and yellow) with 53, 42, 127, 35 coexpressed proteins, respectively. The “gray” module included proteins that were not clustered.

**Figure 1:**
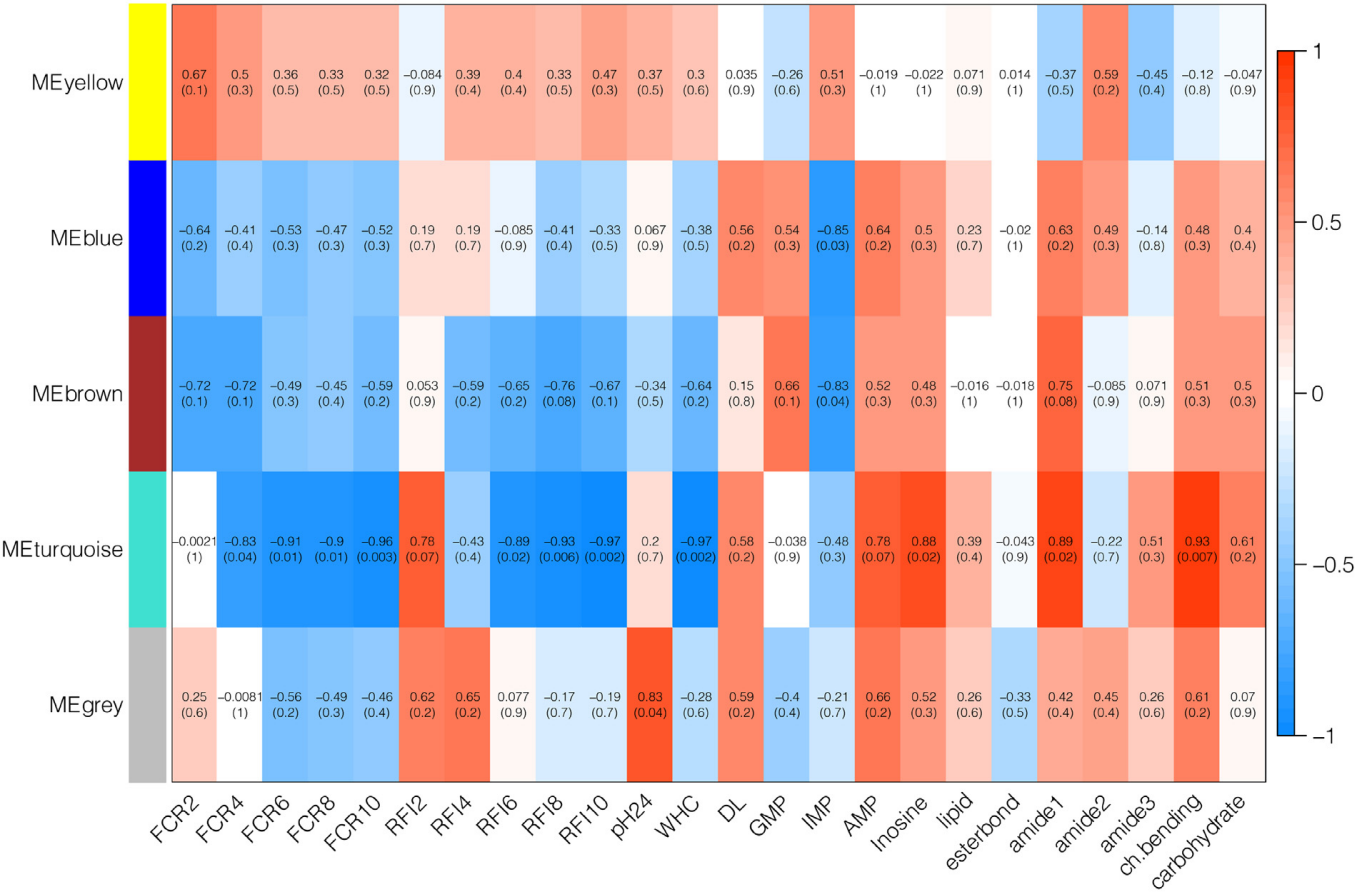
Correlations between protein modules and feed efficiency and meat characteristics traits. The module names are given on the *y*-axis and the traits on the *x*-axis. The table is color-coded by correlation values: blue represents a negative correlation while red represents a positive correlation. The numbers in each cell are correlation coefficients and *P* values (in parentheses). FCR 2, 4, 6, 8, and 10; feed conversion ratio at 2, 4, 6, 8, 10 wk of age; RFI 2, 4, 6, 8, and 10, residual feed intake at 2, 4, 6, 8, and 10 wk of age; WHC, water‐holding capacity; DL, drip loss, GMP, guanosine monophosphate; IMP, inosine monophosphate; AMP, adenosine monophosphate; esterbond, ester carbonyl of phospholipids; ch.bending, C–H bending; and carbohydrate, carbohydrate and glycogen.

The association between protein modules and FE and meat characteristic traits is illustrated in Figure 1. The turquoise module had the highest number of significant associations with FE and meat characteristics (Figure 1). A negative correlation was found between the turquoise module and FCR at 4, 6, 8, and 10 wk of age and between the turquoise module and RFI at 6, 8, and 10 wk of age. Thus, the higher the quantity of the eigenproteins in the turquoise module, the better FCR and RFI values the birds had. In addition, the turquoise module had a negative correlation with WHC and a positive correlation with inosine, amide I, and C–H bending. As the turquoise module showed the strongest association with FE and meat characteristics traits, we focus only on the turquoise module in the subsequent analyses.

### Functional Enrichment Analysis of the Turquoise Module

**Figure 2:**
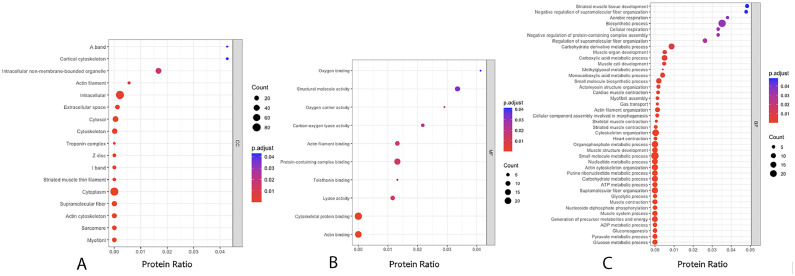
Summary of a functional annotation of the proteins in the turquoise module by gene ontology cellular components (A), molecular functions (B), and biological processes (C). “p. adjust” are the FDR-adjusted *P* values. “count” is the number of proteins enriched in a GO term. “Protein ratio” is defined as the number of proteins associated with particular terms divided by the total number of proteins.

Based on the GO analysis, proteins in the turquoise module were mainly localized in the intracellular (GO:0005622), cytoplasm (GO:0005737), intracellular non–membrane-bounded organelle (GO:0043232), cytosol (GO:0005829), and cytoskeleton (GO:0005856) (Figure 2A). The molecular functions were mainly involved in cytoskeletal protein binding (GO:0008092), actin binding (GO:0003779), protein-containing complex binding (GO:0044877), structural molecule activity (GO:0005198), and lyase activity (GO:0016829) (Figure 2B). The enriched biological processes were small molecule metabolic process (GO:0044281), cytoskeleton organization (GO:0007010), organophosphate metabolic process (GO:0019637), actin cytoskeleton organization (GO:0030036), and carbohydrate metabolic process (GO:0005975) (Figure 2C), which mostly relate to metabolic processes within the muscular cell.

Based on the KEGG analyses, the turquoise module was highly enriched with proteins related to substance or energy metabolism, including the “glycolysis/gluconeogenesis,” “biosynthesis of amino acids,” “pyruvate metabolism,” “metabolic pathway,” “carbon metabolism,” and “protein processing in the endoplasmic reticulum” pathways (Table 3). Our results show that the processes related to energy generation in the thigh muscles of slow-growing chicken are related to FE, muscle metabolism, biomolecules, and flavor precursors and meat characteristics.

**Table 3:** A summary of the KEGG pathways for proteins in the turquoise module.

KEGG ID	Description	Protein count^1^	Adjusted *P* value^2^
00010	Glycolysis/gluconeogenesis	8	3.67E-10
01200	Carbon metabolism	8	3.40E-08
01230	Biosynthesis of amino acids	7	3.40E-08
00620	Pyruvate metabolism	4	8.37E-05
01100	Metabolic pathways	14	8.37E-05
04141	Protein processing in the endoplasmic reticulum	4	0.0159

1 The number of proteins associated with KEGG terms.

2 FDR-adjusted *P* values.

### Candidate Hub Protein Screening

**Figure 3:**
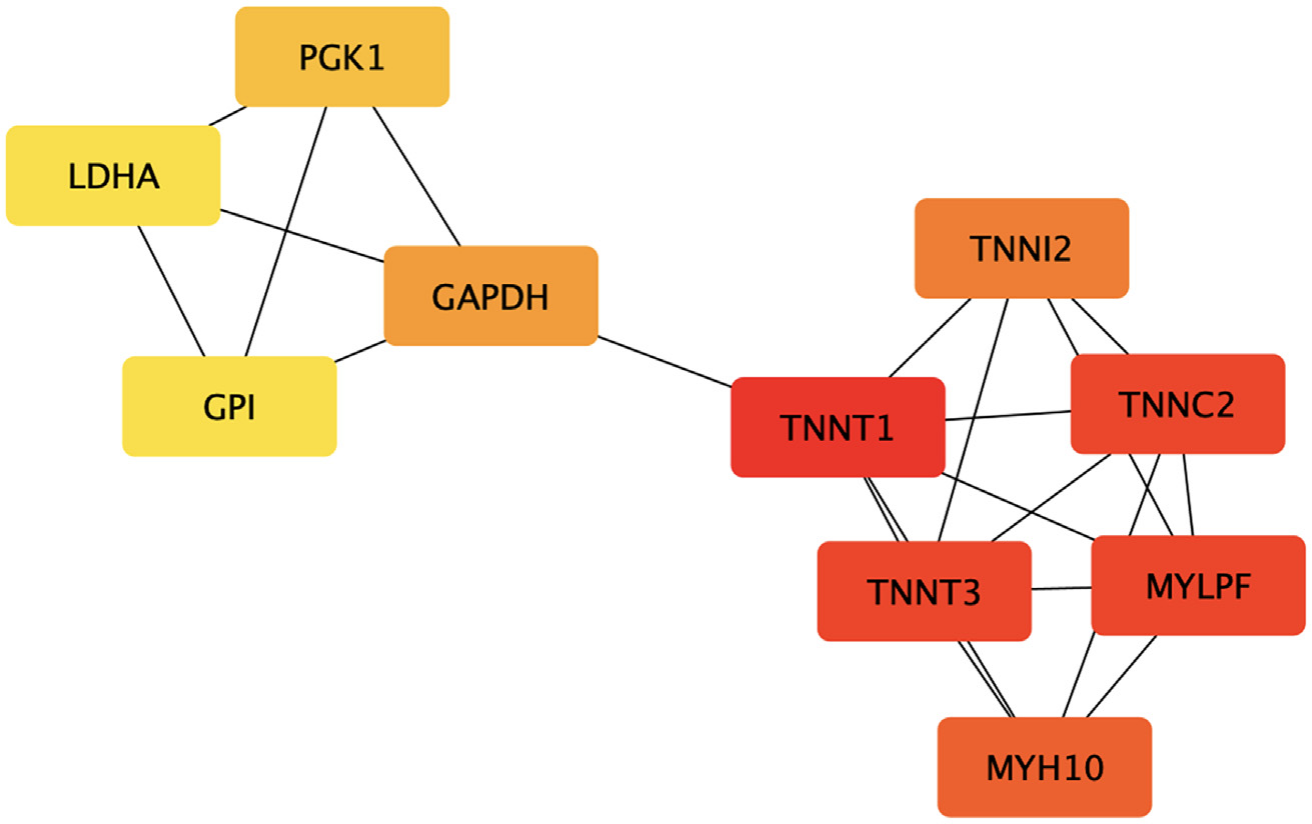
The connectivity plot of the 10 hub proteins using the CytoHubba plugin. The color of a node indicates the degree of connectivity (red for high degree, orange for intermediate degree, and yellow for low degree).

The turquoise module was comprised of 127 proteins (Supplementary Table 2). Among them, proteins that had the strongest correlation (|PS| ≥ 0.6) with FE and meat characteristics traits and the module eigenproteins (|MM| ≥ 0.7) were identified as candidate hub proteins (Supplementary Table 3). Pathway enrichment was further conducted to understand the biological role of these identified hub proteins. Cytohubba revealed 10 hub proteins that were highly connected: **TNNT1** (slow skeletal muscle TnT), **TNNT3** (fast skeletal muscle TnT), **TNNI2** (Troponin I2), **TNNC2** (Troponin C), **MYLPF** (myosin light chain, fast skeletal muscle), **MYH10** (Myosin Heavy Chain 10), **GADPH** (glyceraldehyde-3-phosphate dehydrogenase), **PGK1** (phosphoglycerate kinase), **LDHA** (L-lactate dehydrogenase A chain), and **GPI** (glucose-6-phosphate isomerase) (Figure 3). These proteins were considered key regulators for both FE and meat characteristics.

## DISCUSSION

Korat chicken is a Thai crossbreed that is known for its high meat quality but with a relatively low FE. Improving FE and meat characteristics is thus crucial for profitable and competitive chicken production. The association between FE and meat characteristics is poorly understood at the proteomic level. Our research is the first to explore the role of key regulator proteins and pathways underlying FCR and RFI at various development stages and meat characteristics such as the physicochemical properties, flavor indicators, and biomolecules of Korat chicken thigh muscles.

We presented 3 main hypotheses: 1) the regulation of FCR and RFI depend on several molecular and physiological mechanisms, 2) a negative relationship exists between FE and meat characteristics, and 3) the molecular and physiological determinants of FCR and RFI are age dependent. Surprisingly, our results did not support the first hypothesis, only one module (turquoise) was associated with FCR and RFI. Pathways related to proteins of the significant module, including glycolysis and gluconeogenesis, metabolic pathway, carbon metabolism, amino acid biosynthesis, pyruvate metabolism, and protein processing in the endoplasmic reticulum (**ER**), are part of the complex biological events of protein biosynthesis, energy generation, and energy storage in the skeletal muscle.

Glycolysis/gluconeogenesis was previously reported to be one of the significant pathways associated with FE (Abasht et al., 2019; Kaewsatuan et al., 2022). Glycolysis is the cytoplasmic pathway that efficiently produces ATP (energy) from glucose by converting it into 2 pyruvate molecules. Pyruvate can be completely oxidized in the mitochondria to generate ATP via the tricarboxylic acid cycle (**TCA**) and oxidative phosphorylation, while gluconeogenesis is the metabolic process through which glucose is synthesized from noncarbohydrate precursors. These pathways play a key role in regulating the glucose and energy homeostasis of tissues with high metabolic demand, such as the skeletal muscle (Abasht et al., 2019).

Moreover, the biosynthesis of amino acids and protein processing in the endoplasmic reticulum pathways were observed to have a significant effect on the FE of animals (Lee et al., 2015; Pezeshkian et al., 2022). Amino acids are fundamental components of body proteins. Once amino acids enter a cell, they can be used as precursors for energy production or used for other biochemical processes, such as protein synthesis, depending on metabolic requirements (White et al., 2021). Protein synthesis is primarily regulated at the initiation phase of protein translation in the cytosol and ER compartment (Stephan et al., 2005). The ER is the main organelle responsible for calcium homeostasis, quality control, protein synthesis, and protein folding. The accumulation of unfolded or misfolded proteins causes stress to the ER (Marques et al., 2021). An increase in this pathway possibly increases the ER's capacity for facilitating protein folding and synthesis to restore protein homeostasis.

Selecting for lower FCR increases body weight gain and leads to slightly higher feed intake (Yi et al., 2018). One possible explanation for the relationship between energy metabolism-related pathways, protein synthesis, and FCR may be that a weight gain typically leads to changes in body composition, including a significant increase in metabolic tissues (e.g., muscle protein mass), which contributes to energy use in animals (Galgani and Ravussin, 2008). The increase in body weight is generally due to an increase in muscle fiber size (hypertrophy) and a greater number of muscle fibers (hyperplasia) (Wang et al., 2020b). The rate of muscle protein accumulation depends on the balance between energy intake and expenditure; the excess of substrate and energy intake lead to an increase in muscle protein deposition (Van Milgen et al., 2001). Meanwhile, selecting for RFI reportedly associated with differences in feed intake, feed digestion, metabolism, and thermoregulation (Yi et al., 2018; Yang et al., 2020; Xiao et al., 2021). As feed intake increases, a larger amount of energy is required to supply for the digestion processes in terms of increased digestive organ size and increased energy consumed for supporting tissue activities. Thus, an animal with a lower feed intake is expected to be more efficient because less energy is expended on tissue maintenance and activities (Yang et al., 2020; Xiao et al., 2021).

Our results showed that the turquoise module was negatively associated with FCR and RFI, indicating that greater efficiency in high-FE chicken may be due to an increased rate of glycolytic potential and protein-synthesizing potential. Efficient animals reportedly accumulate more muscle mass than inefficient animals (Zhuo et al., 2015). High-FE chickens are speculated to use more glucose to generate ATP for protein deposition and for maintaining tissue homeostasis.

In addition to the negative correlation between the turquoise module and FE, we observed significant correlations between the turquoise module and certain meat characteristic parameters; a negative correlation with WHC and positive correlations with inosine, amide I, and C–H bending. Given that the same proteins and pathways are correlated with FE and meat characteristics but in opposite directions, changes in FE can negatively impact the meat characteristics, flavor, and biomolecules in thigh meat through alterations to metabolic processes, that is, glycolysis and gluconeogenesis, metabolic pathway, carbon metabolism, amino acid biosynthesis, pyruvate metabolism, and protein processing in the ER. These findings are in line with our second hypothesis. The potential role of the discovered pathways affecting meat characteristics is discussed below.

Immediately after slaughter, the muscle temporarily converts pyruvate into lactate to maintain the homeostasis of ATP concentration when oxygen is limited. During the postmortem period, ATP can be degraded into adenosine diphosphate (**ADP**), adenosine monophosphate (**AMP**), inosine monophosphate (**IMP**), and other derivative compounds. IMP gradually changed into inosine, causing a bitter taste in the meat (Ishiwatari et al., 2013; Dashdorj et al., 2015). Thus, the increased activity of the pyruvate metabolism may be a key factor in promoting inosine accumulation in the thigh meat of high-FE chicken and may cause unfavorable flavor in the meat.

The elevation of glycolysis and pyruvate metabolism result in the accumulation of lactic acid, which consequently reduces the pH value of the postmortem meat (Vanderhout et al., 2022). As the process of protein folding is critical to the proper functioning of the muscle protein, any disruptions in this process can lead to misfolded structures that may impact the quality of meat, for example, a rapid and extensive pH decline increases the denatured muscle proteins and impairs the functionality of meat (Stajkovic et al., 2019). An increase in the amide I (1,700–1,600 cm^−1^) integral area may have resulted from a higher protein content in the meat. Additionally, the high-FE group had a higher integral area of C–H bending at 1,450 and 1,380 cm^−1^. These changes have been suggested to result from exposure of the aliphatic hydrophobic side chain of proteins to the aqueous environment (Katemala et al., 2022; Suwanvichanee et al., 2022). The stability of the protein structure depends on various interactions, including covalent and hydrogen bonds, hydrophobic interactions, electrostatic, and Van der Waals forces (Herrero, 2008). Alterations in pH levels can disturb these interactions and hydrophobic forces, leading to the loss of their tertiary and secondary structures and hydrophobic amino acid groups getting exposed (Puolanne and Halonen, 2010). Based on our findings, we present the following hypothesis: an increase in the integral areas of amide I and C–H bending causes a higher hydrophobic residue content in the protein structure in the thigh meat of high-FE chickens. These changes may reduce the number of charged protein sites for binding water, leading to a decrease in WHC, and consequently an undesirable texture of the meat. Therefore, our findings indicate that the improvement of FE could potentially have an unfavorable effect on the texture and flavor of meat. Again, these results are in line with our second hypothesis.

In addition to glycolysis/gluconeogenesis and the protein-synthesizing pathways that were identified, mitochondrial activity has previously been reported to be among the most significant pathways influencing FE (Bottje et al., 2002; Kong et al., 2016; Yang et al., 2020) and meat quality (Abaht et al., 2019; Bottje et al., 2021). Mitochondria function is critical for improving FE due to its potential role in generating and utilizing energy extracted from feed (Bottje et al., 2006). One of our research objectives was to identify the shared pathways that were linked with both FE and meat quality traits. Although we found proteins related to mitochondrial oxidative phosphorylation that are associated with FE, the same proteins did not affect the quality of meat. It is possible that the role of mitochondria in meat texture is indirect because their role is not limited to energy generation, as these organelles also participate in diverse cellular processes like calcium signaling, apoptosis, and oxidative stress (Dang et al., 2020). Further studies focused on mitochondria would be needed to determine the underlying causes.

Of the obtained hub proteins, TNNT1, TNNT3, TNNI2, TNNC2, MYLPF, MYH10, GADPH, PGK1, LDHA, and GPI showed high degrees of connectivity and can be considered key regulators of both FE and meat characteristics (Figure 3), that was previously observed in a transcriptome study of broiler breast muscle (Kong et al., 2011; Bottje et al., 2017). TNNT1 (slow skeletal muscle TnT) and TNNT3 (fast skeletal muscle TnT) are the tropomyosin-binding subunits that comprise the elongated portion of the troponin complex (Tn-complex) (Marston and Zamora, 2020). **TNNI2** (Troponin I2; TnI) is the inhibitory subunit of the Tn-complex, and **TNNC2** (Troponin C) is the Ca^2+^-binding subunit of the Tn-complex. These 3 subunits compose the Tn-complex structure, which has an important role in the calcium-dependent regulation of skeletal muscle contraction and relaxation (Wei and Jin, 2016). Mutations in the 3 TnT isoform genes (i.e., *TNNT1, TNNT2*, and *TNNT3*) have been found in cardiac and skeletal myopathies, suggesting that TnT plays a key role in striated muscle growth and function (Sheng and Jin, 2014).

**MYLPF** (myosin light chain, fast skeletal muscle) is reportedly associated with skeletal muscle tissue development (Wang et al., 2007). In zebrafish (*Danio rerio*), knockout of *mylpfa* causes degeneration of differentiated skeletal myofibers (Chong et al., 2020). The MYH10 protein (Myosin Heavy Chain 10) regulates cytokinesis, cell motility, and cell polarity (Ridge et al., 2017). PGK1 (phosphoglycerate kinase) is known for the first ATP-yielding step via a PGK-catalyzed reaction, which participates in the reversible reaction from 1,3-bisphosphoglycerat to ADP to form 3-phosphoglycerate and ATP, catalyzed by phosphoglycerate kinase (Alto et al., 2021). Kierans and Taylor (2021) reported that the *PGK 1* gene was upregulated under hypoxia conditions, suggesting that an increase in glycolytic activity during hypoxia stress may help preserve bioenergetic balance. Glyceraldehyde-3-phosphate dehydrogenase (**GAPDH**) is an anaerobic regulator of glycolysis and is widely used as an internal control when comparing gene and protein expression levels. It catalyzes the reversible conversion of glyceraldehyde-3-phosphate to 1,3-diphosphoglycerate during glycolysis (Wang et al., 2020a). GPI catalyzes the reversible reaction of glucose-6-phosphate to fructose-6-phosphate (Haller et al., 2011). Knocking down *GPI* gene expression can decrease the glycolytic gene activity and endogenous glucose level in PGCs in the primordial germ cells (**PGCs**) of chicken (Rengaraj et al., 2012). Additionally, lactate dehydrogenase A (**LDHA**) catalyzes the reversible conversion of pyruvate to lactate and acts as an indicator of glycolytic capacity (Zhao et al., 2020). LDHA reportedly plays a key role in lactate homeostasis and energy balance and was previously identified as a candidate gene for muscle development (Qiu et al., 2010). Because of their functions, these discovered hub proteins may potentially affect FE and meat characteristics.

The turquoise module correlated significantly with FCR for wk 4 to 10 and with RFI for wk 6 to 10 (Figure 1 and Supplementary Table 3), indicating age dependency and supporting our third hypothesis. Consistently, Liu et al. (2016) reported that pathways involved in muscle development, including hypertrophic cardiomyopathy, cardiac muscle contraction, tight junctions, and focal adhesion, were prominent between d 56 and 98 in Beijing-You chicken. However, it is necessary to examine the expression of these 10 hub proteins at various ages to deepen our understanding of the molecular mechanism underlying age-dependent FE.

## CONCLUSIONS

To our knowledge, this is the first study that analyzes the complex protein regulation system underlying FE and meat characteristics in a slow-growing chicken breed. In this study, WGCNA was applied to detect the key proteins affecting both FE and meat characteristics using proteomic data of the thigh muscle. The 10 most important hub proteins obtained (TNNT1, TNNT3, TNNI2, TNNC2, MYLPF, MYH10, GADPH, PGK1, LDHA, and GPI) play a critical role in molecular responses involved in energy metabolism and muscle development. In addition, our findings provide evidence that improving FE may have an unfavorable effect on the texture and flavor of thigh meat, which should be accounted for in the selection programs of meat-producing chicken breeds.

## Appendix Supplementary materials

Image, application 1
